# Brief meditation increases fiber wiring between striatum and corona radiata

**DOI:** 10.1186/1471-2202-15-S1-P33

**Published:** 2014-07-21

**Authors:** Yi-Yuan Tang, Huiyan Shao, Rongxiang Tang

**Affiliations:** 1 Department of Psychology, Texas Tech University, Lubbock, TX 79409, USA; 2 Department of Psychology, University of Texas at Austin, Austin, TX 78705, USA

## 

White matter tractography has becoming increasingly useful for the visualization and measurement of brain anatomical structures and connections in both cortical and subcortical regions. Our previous studies using Diffusion Tensor Imaging have revealed the changes of white matter indexes including fractional anisotropy, axial diffusivity, and radial diffusivity at the corona radiata, an important white matter tract connecting the anterior cingulate cortex (ACC) to other structures including striatum after brief meditation training - integrative body-mind training (IBMT) in healthy college students [1,2]. Since ACC and striatum involve in self-control networks, here we seek to further investigate the mechanism of white matter changes by employing an innovative tracking method and targeting specific fiber bundle connecting ACC and striatum after 2-week of IBMT.

Fourteen healthy undergraduates were recruited at campus and received 30 min of IBMT for 2-wk, with a total of five hours training. IBMT involves body relaxation, mental imagery, and mindfulness training, accompanied by selected music background and guided by IBMT coach [1,2]. DTI scans were preformed before and after 2-wk IBMT with a Philips 3T Achieva. DTIstudio was used for preprocessing that includes motion and eddy current correction, coregistration, and subsequent calculation of diffusion tensors and FA maps. Fiber Assignment by Continuous Tractography (FACT) method was then used with FA threshold set at 0.2 and principal eigenvector set at 45° for fiber tracking. Normalization was carried out with DiffeoMap to normalize subject images to atlas by linear and non-linear transformation. With the inverse linear and non-linear transformation matrices, the atlas, a brain parcellation map with 130 segmented ROI objects, was warped to the individual data space for each subject. ROIs in the left anterior corona radiata (ACR) and striatum were then defined by ROIeditor [3]. We focused on the number of fibers for the fiber bundle between striatum and left ACR, and compared within-group changes before and after 2-wk training. We found increased number of fibers penetrating both striatum and left ACR after 2-wk IBMT (P=0.000).

## Conclusion

Our results indicated brief meditation increases fiber wiring between ACR and striatum, suggesting the improved connectivity between self-control and reward networks. These may provide neurobiological base for positive behaviour, habit and characters.

**Figure 1:**
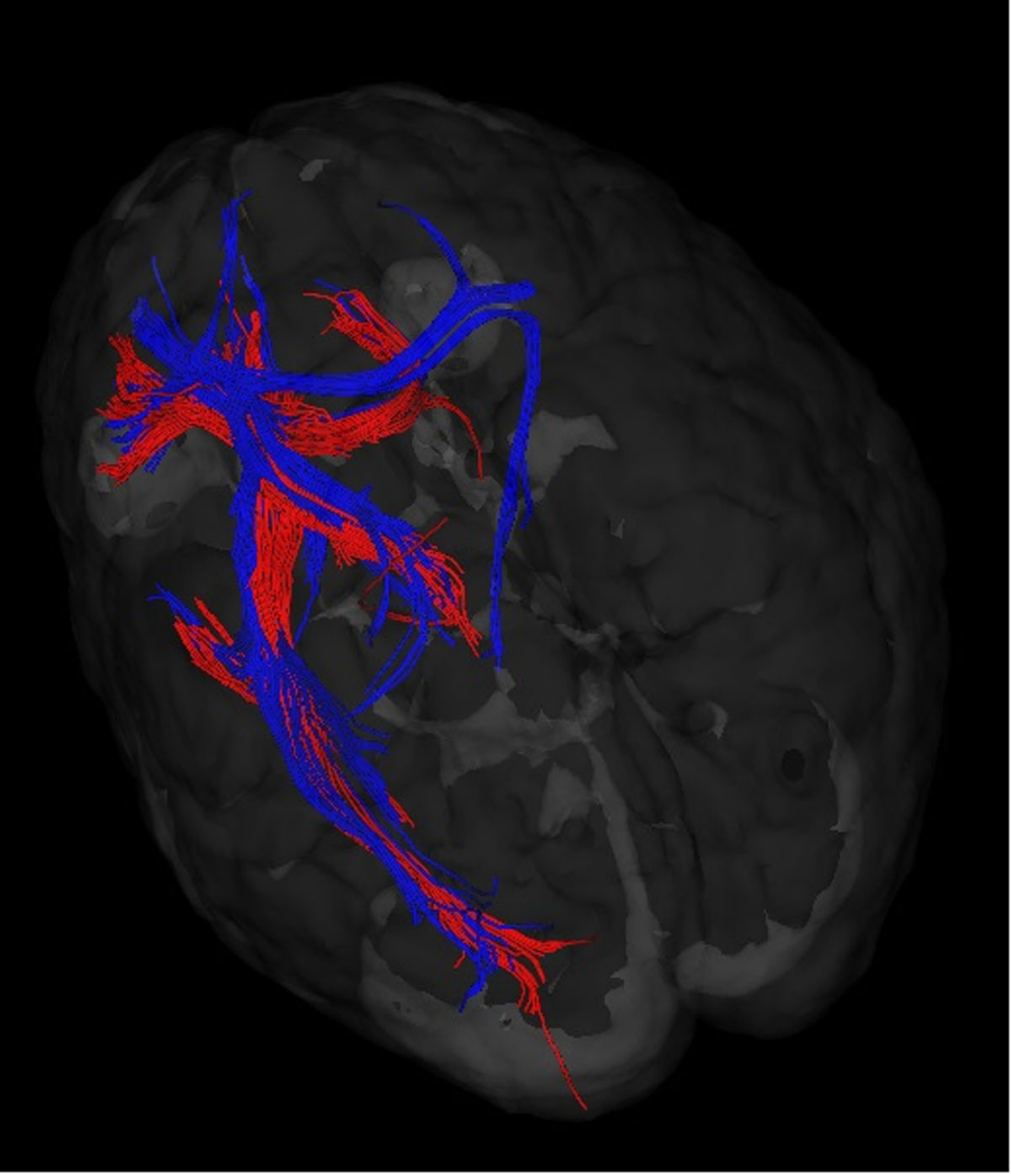
Fiber results of a single subject. Red represents pre-training data, blue indicates 2-wk training data. Fiber penetrating both striatum and left ACR is shown here.
